# Measuring spatial accessibility to refuge green space after earthquakes: A case study of Nanjing, China

**DOI:** 10.1371/journal.pone.0270035

**Published:** 2022-06-28

**Authors:** Wei Liu, Hao Xu, Jing Wu, Wei Li, Huimin Hu

**Affiliations:** 1 College of Landscape Architecture, Nanjing Forestry University, Nanjing, PR China; 2 Key Laboratory of Landscape Architecture of Jiangsu Province, Nanjing, PR China; Public Library of Science, UNITED STATES

## Abstract

The construction of refuge spaces in rapidly urbanizing historic cities is a challenging task owing to their complex urban form, unique urban fabric, and historic preservation requirements. Refuge green space (RGS) is a green space that can serve as an emergency shelter in cities, providing a flexible means to increase the emergency shelter capacity for rapidly urbanized historic cities. After major earthquakes, spatial accessibility to GRSs is a critical planning strategy for disaster prevention and emergency response in historic cities. To match the RGS planning with the emergency service demand, we must examine the spatial disparity in access to existing RGSs. In this study, the urban area of Nanjing was selected as the target region to analyze the spatial disparity in access to RGSs using the Gaussian two-step floating catchment area method at four evacuation times (10, 20, 30, and 60 min). The results showed that the spatial accessibility exhibited clustering characteristics, where high-accessibility spaces were mainly distributed in the northern and southern regions of Nanjing. The increase in the evacuation time improved accessibility to RGSs, but the existing RGSs still could not sufficiently satisfy the emergency shelter needs of citizens. Based on the bivariate local *Moran’s I* analysis of the RGS accessibility and population density, the spatial mismatch regions were dominant in the center of the urban area. These findings not only are expected to assist emergency planners by improving their strategic plans for emergency shelter investment in Nanjing and their ability to respond to catastrophic earthquakes, but also provide a strong reference for the construction of a safe environment in other rapidly developing historic cities that face earthquake threats.

## Introduction

China is an earthquake-prone country and has experienced approximately 20 major earthquakes since the beginning of the 20th century, such as the Tangshan, Wenchuan, and Yushu earthquakes, which have killed hundreds of thousands of people and resulted in hundreds of billions of yuan in damage [1]. High-magnitude earthquakes that occur in developing countries undergoing urbanization or in densely populated areas will cause more catastrophic damage and destruction [2]. Owing to the sudden and rapid occurrence of earthquakes, accurately predicting the timing, location, and magnitude of earthquakes is highly difficult. Emergency evacuation and resident resettlement are critical post-earthquake actions for reducing the loss of life, which is the priority after an earthquake [3]. Therefore, emergency shelters should be planned in advance to achieve rapid post-earthquake recovery.

Historic cities, however, are among the most vulnerable to disasters [4, 5], particularly in a rapidly urbanized developing countries subject to earthquakes, similar to those occur in China. Complex morphologies current cities are a natural result of centuries-old development processes. Considering the need to protect historic values, building emergency shelters by changing the existing fabric of a historic city to secure residential safety is highly difficult. Therefore, coordinating the relationship between rapid urbanization and emergency shelters, as well as enhancing the capacity of urban emergency services, is one of the major challenges for historic cities.

Refuge green spaces (RGSs) are urban parks, attached green spaces, public squares, and playgrounds with basic emergency services (e.g., first aid stations) and utilities (fresh water, electricity, and communication systems) [6, 7]. During daily life, these spaces are used for recreation, sightseeing, cultural activities, and exercise. When disasters occur, they serve as emergency shelters. RGSs are ideal emergency shelters, because they not only provide space for evacuation and temporary transfers, but also prevent secondary disasters after a major earthquake. Furthermore, RGSs provide an opportunity to enhance the overall quality of the urban environment [8], which allows for a flexible means to increase the number of emergency shelters in rapidly urbanized historic cities. RGSs have provided refuge for historic cities during several large earthquakes [9]. For example, after the Great Kanto earthquake of Japan, approximately 70% of residents chose to take refuge in green spaces [10]. To improve disaster preparedness and emergency shelter planning, many countries have incorporated RGSs into their disaster prevention systems, such as the City Park Law and Preservation of Urban Green Space Law in Japan, Evaluation and Shelter Guidance in the UK, and the National Response Framework in the United States [11–13].

The selection of an appropriate RGS location is critical for the rapid evacuation of citizens after a major disaster [7, 14]. Recent research on RGSs has mainly focused on site reconstruction, infrastructure supply, and service effectiveness [6, 15, 16], and the last is the core of an RGS establishment [17]. In China, rough estimates of the population density of the corresponding subdistrict often determine the scale of emergency shelters. A capability parameter of at least 1.0 m^2^ per person [18] is inflexibly used for RGS planning. However, meeting the indicator requirement does not necessarily suggest that the emergency shelter services are strong and sufficient. The actual effect of the RGS shelters usually differs from the emergency plans.

Spatial accessibility is a combined measure of the availability of local supply points (e.g., RGSs) and accessibility (distance and time) between a demand point (e.g., settlement or, house) and a supply point [19]. It is an important indicator of the relationship between the service effectiveness of public facilities and population demand [20]. Most studies on RGS accessibility have only employed statistical indices at an area scale, such as the total shelter service area, service population ratio, and shelter area per capita [21]. However, the size and service population not only influences RGS accessibility, but also by the RGS distribution and road network. With the development of geographic information system (GIS) technology, some studies have quantified accessibility indicators by building mathematical models based on the evacuation distance or time [6, 22]. These models, such as gravity models, network analysis methods, and two-step floating catchment analysis (2SFCA) methods, consider the road network and spatial interactions between RGS supply and demand to more accurately quantify spatial accessibility [23, 24]. One of the most popular approaches is the 2SFCA method, which was first used to measure medical accessibility in Illinois (based on the physician-to-population ratio metric) and identify areas with limited healthcare services [25]. Recently, the 2SFCA method has been used to evaluate the spatial accessibility of the service levels of public facilities [26–28]. However, the traditional 2SFCA method has several limitations: it neglects distance attenuation, uses the Euclidean distance between the supply and demand points instead of the actual distance, and is built on a fixed catchment size [25, 29]. To address these drawbacks, the Gaussian function was incorporated into the 2SFCA model. Here, the accessibility results showed not only attenuation, but also larger areas of poor accessibility than the 2SFCA [30, 31]. The Gaussian-based 2SFCA (Ga2SFCA) method has been successfully used to evaluate the spatial accessibility to emergency shelters and open spaces [22, 28].

Compared with new development cities, historic cities, especially those that have experienced rapid urbanization, are more prone to the devastating effects of a strong earthquake, resulting in considerable casualties. Therefore, objectively evaluating the accessibility of RGSs in historic cities and their potential of providing evacuation is an urgent task. Studies on the accessibility of refuge space accessibility have selected street- or block-scale census data for visualization, ignored spatial variations within street/block units, and roughly summarized geographic disparities with respect to RGS access [17, 32, 33]. In addition, most studies have used open spaces with potential refuge services instead of existing RGS [6]. These results therefore provided limited support for government policies. In this study, we selected existing RGSs in Nanjing as the study object. Nanjing, as one of the fastest growing cities in China, is a famous historic city. However, it has been suffered the effects of many earthquakes of different magnitudes. The rapidly increasing population, continued expansion of construction land, and limited evacuation space have limited Nanjing’s ability to provide emergency services after a major earthquake. Recently, Nanjing has attempted build RGSs. Since the establishment of the first refuge park (i.e., the “National Defense Refuge Park”) in 2010, 120 RGSs have been built in Nanjing. Whether these RGSs can meet the emergency shelter needs of residents and how to fairly allocate emergency shelters in the future have become concerns for local/national governments and researchers. In this study, the Ga2SFCA method was used to analyze the accessibility of existing RGSs in the urban area of Nanjing. The spatial relationship between accessibility and population density was explored to provide recommendations for the selection and construction of emergency shelters for future disaster preparedness in Nanjing.

The novelty of this study lies in its focus on the construction of RGSs in rapidly urbanizing historic cities. Awareness of the vulnerability and exposure of historic cities to seismic hazards has initiated research on refuge spaces and emergency management [4, 5]. However, few studies have examined the effectiveness of refuge spaces in rapidly developing historic cities, where their irreplaceable historic fabric is often neglected during refuge space construction. Our research explores the accessibility of RGSs using well-established methodologies, to propose policy implications for emergency plans based our findings, which are not only beneficial to Nanjing, but also other rapidly urbanizing historic cities throughout the world.

## Materials and methods

### Study area

Nanjing is the capital of Jiangsu Province of China, located downstream of the Yangtze River, ranking in the first tier of Chinese cities in terms of its economic development and urban construction. It is the second largest city in eastern China, with an urbanization rate of > 80%. However, the city is not only characterized by a flourishing economy, but it also has an urban history of spanning 2,500 years. Having been the capital city of six different Chinese dynasties, Nanjing has abundant cultural and architectural heritage, such as the Presidential Palace, Sun. Yat-sen Mausoleum, and Ming Palace.

For its geological structure, Nanjing is located in the Yellow Sea seismic zone, which is characterized by high-frequency seismic activity and in situ recurrence characteristics [34]. This zone possesses the geological conditions for moderate and strong earthquakes, because there are four hidden faults with a total length of approximately 380 km, whose pose a significant threat to Nanjing [34, 35]. Historically, Nanjing has been struck by many earthquakes of different magnitudes: a series of 42 earthquakes occurred in 1425 [36]. The sound of some earthquakes was described to be as loud as thunder, which implies that they were large-magnitude earthquakes. In 1624, a 6.0 magnitude earthquake caused severe damage [37]. Recently, a number of small earthquakes have been detected [38], some of which have disrupted daily life. Remarkably, research on the characteristics of Quaternary fault activity around the Nanjing area has shown that there is the potential for an earthquake with a maximum magnitude of 6.0 [39]. The city has recently initialized guideline, i.e., the “Nanjing earthquake emergency shelter evacuation and resettlement” to further strengthen their emergency evacuation capabilities, which aim to respond to moderate-to-high intensity earthquakes [40].

**Fig 1:**
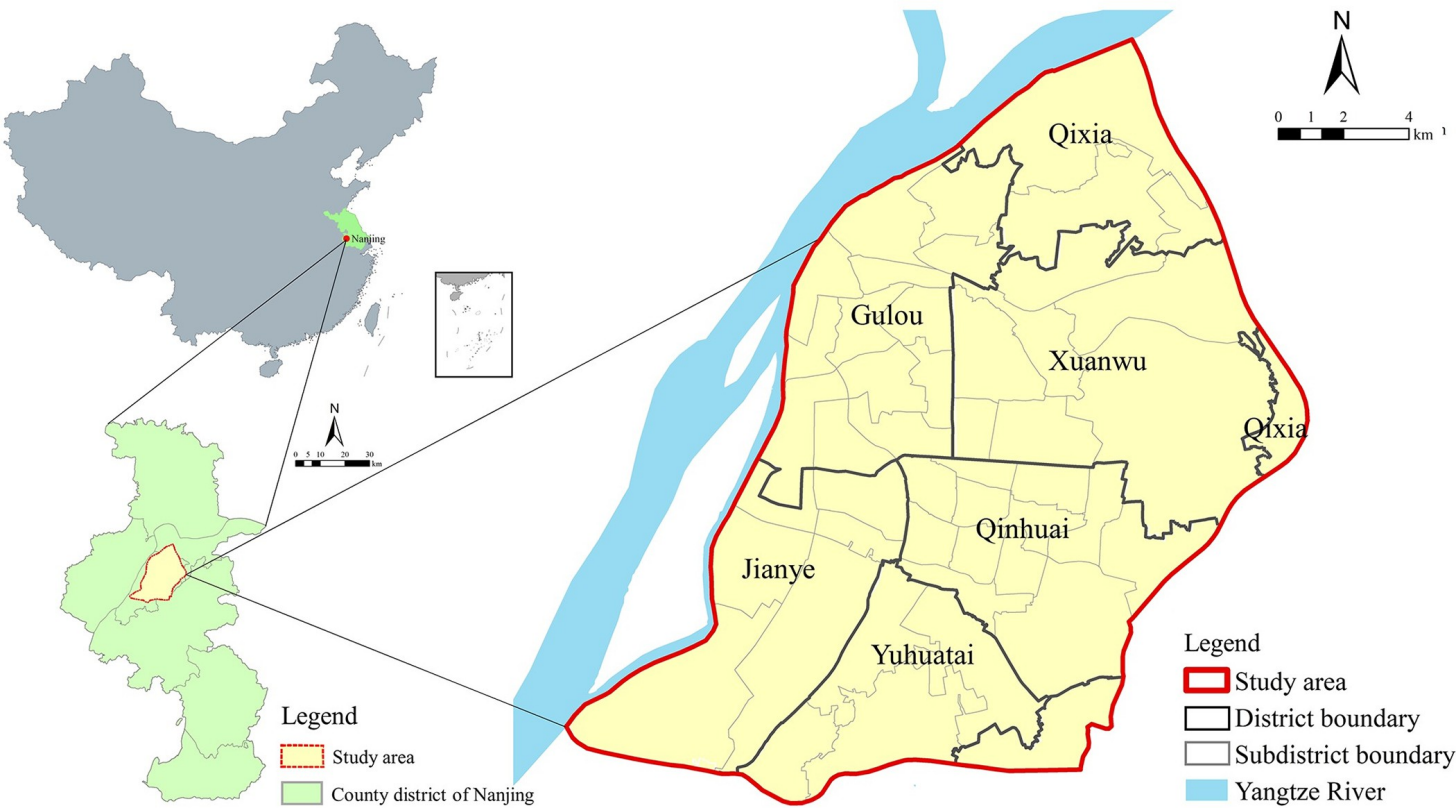
Location of the study area.

The urban area of Nanjing, which is bounded by the Yangtze River, Qinhuai New River, and bypass highways (Fig 1), has a relatively complete urban structure within the ancient capital. It is also the most densely populated and built-up area in the city. A damaging earthquake could place an enormous amount of stress on the evacuation system of its urban spaces. Therefore, the urban area of Nanjing, with a total area of approximately 280 km^2^, including most of the Gulou, Xuanwu, and Qinhuai districts, as well as some parts of the Jianye, Yuhuatai, and Qixia districts, was selected to evaluate RGS accessibility.

### Data collection

In this study, 2019 data on the names, locations, and effective refuge areas for RGSs were collected from the Nanjing Emergency Management Bureau (http://yjzh.njyjgl.cn:8000/#/). The 2019 annual demographic statistics were obtained from the Nanjing Statistics Bureau. Road network data were obtained from OpenStreetMap (https://www.openstreetmap.org). In previous studies, green spaces centroids were typically considered target sites to measure the RGS accessibility [22]. However, RGSs have many entrances, such that the results of an accessibility analysis would be more accurate than those calculated by only considering green spaces centroids. Hence, entrance sites for RGSs determined using portable high-accuracy GPS devices were selected for further analysis.

**Fig 2:**
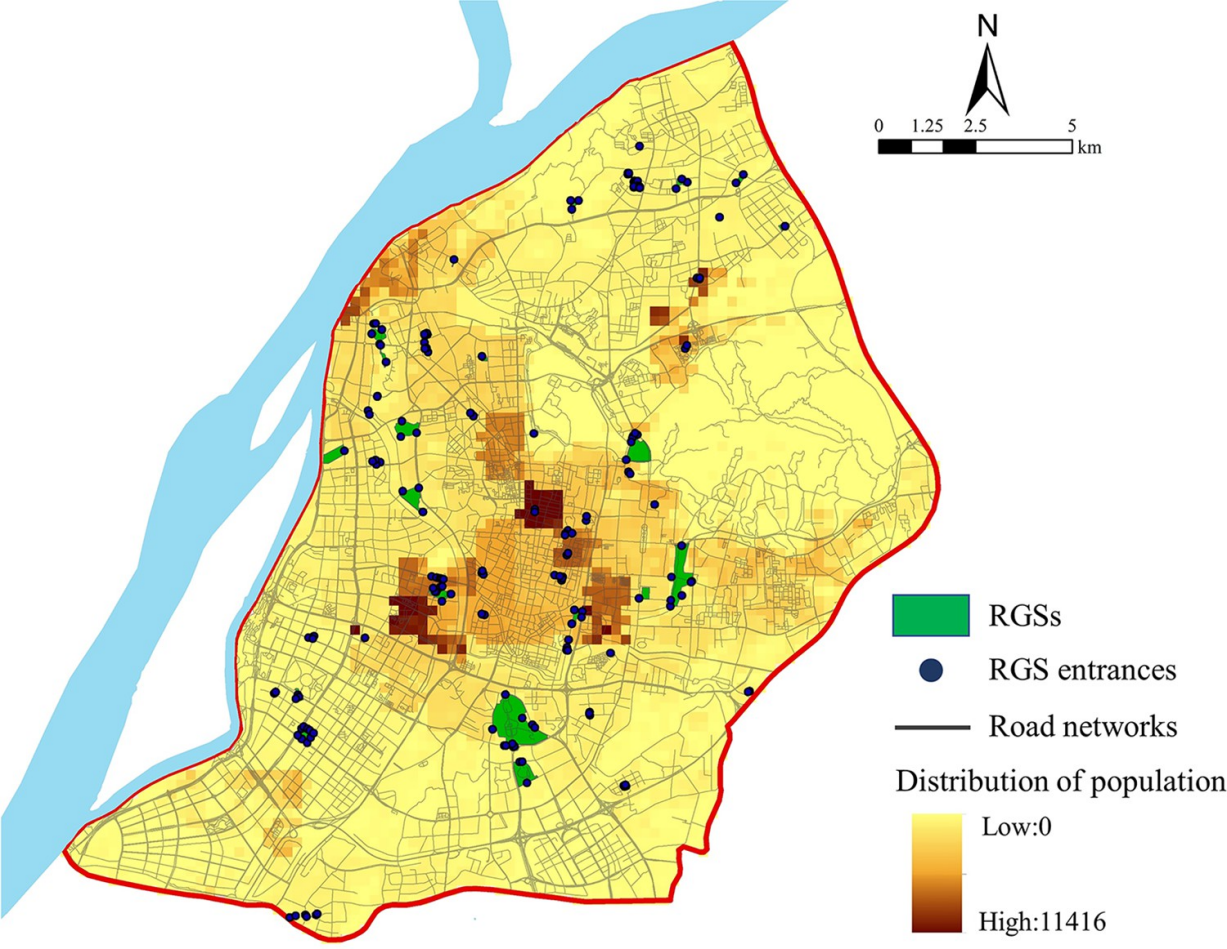
Dataset on the emergency open space (RGS) accessibility in Nanjing.

The subdistrict is the minimum spatial unit in the Chinese population census. Its centroid is generally regarded as the population demand location of refugees living in this unit. However, the shapes of most subdistricts are irregular, which can lead to more significant errors in the spatial accessibility based on a centroid analysis. Population data grid transform is an effective manner of reducing the calculation error based on the irregularities of subdistricts [41]. It has been applied in other studies on the accessibility of public facilities [22, 42, 43]. In this study, each subdistrict was divided into 250 m × 250 m cells, and the population data of each subdistrict were allocated to the corresponding cells according to the building density. A total of 4666 spatial cells were obtained in the entire study region, and the geometric center of each cell was assumed to be the population demand location. Finally, the road network of the Nanjing urban area, 57 RGSs, 174 RGS entrances, and 4,666 population demand locations were used to explore the accessibility of RGSs in Nanjing (Fig 2).

## Methodology

**Fig 3:**
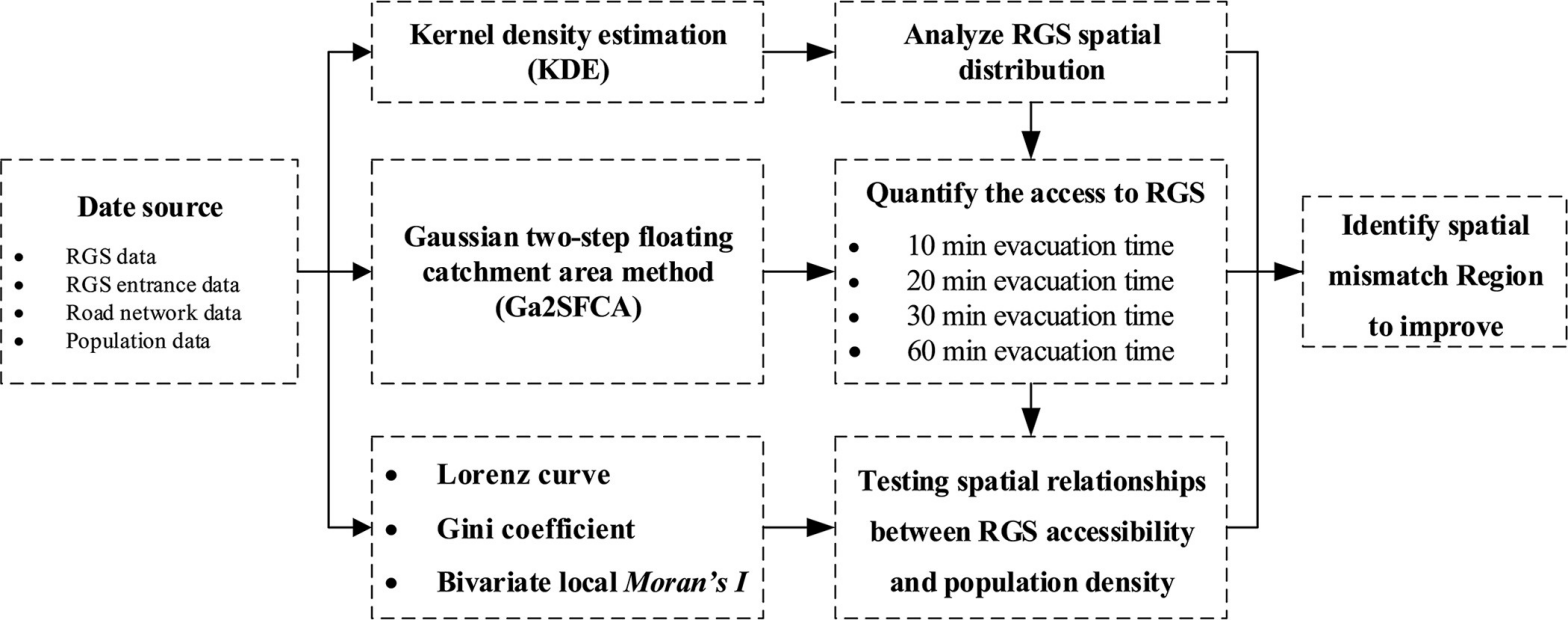
Flowchart of the study.

The flowchart in Fig 3 displays the methods used in the study. First, kernel density estimation (KDE) method was used to analyze the spatial patterns of existing RGSs in the study area. Ga2SFCA was then employed to acquire the spatial accessibility of RGSs, and explore the variability in accessibility under four evacuation time scenarios. Finally, the Lorenz curve, Gini coefficient, and bivariate local *Moran’s I* were used to explore the spatial relationships between the RGS accessibility and population density in Nanjing and identify areas of mismatch between the RGSs and population. The following sections more details on the specific methods.

### Kernel density estimation

Kernel density estimation (KDE) estimates the underlying probability density of a dataset and obtains a continuous and smooth density distribution map of spatial elements through a specific radius input [44]. KDE is widely used to examine the spatial distribution of service facilities [45]. In this study, the RGS spatial distribution density was calculated based on the KDE tool in the ArcGIS software platform, thus allowing for the further analysis of the heterogeneity in the RGS spatial distribution in the study area. The radius was automatically defined and the quantitative classification used the Jenks natural breaks method.

### Ga2SFCA method

Ga2SFCA has been successfully applied in green space and refuge space accessibility field, because the calculation results more accurately reflect reality [22, 28]. Therefore, Ga2SFCA was chosen for RGS accessibility measurements in this study. The specific calculation steps were as follows.

First, for each supply location (e.g., RGS) (j), we searched all of the demand locations of the population (*k*) within the evacuation distance (catchment area j) and then computed the supply-to-demand ratio (*R*_*j*_), which is the ratio of the effective refuge areas at location *j* to the total population after a distance discount is applied within catchment area *j*:.

$$R_{j}=\frac{S_{j}}{\underset{k\in \{d_{kj}\leq d_{o}\}}{\sum }[g(d_{kj},d_{o})P_{k}]},$$ (1)

where *S*_*j*_ is the effective refuge area of *j*, *P*_*k*_ is the population of *k* whose centroid is located within the catchment area *j* (*d*_*kj*_≤*d*_*o*_), *d*_*kj*_ is the evacuation distance between *k* and *j*, d_o_ is the threshold of evacuation distance, and *g*(*d*_*kj*_, *d*_*o*_) is the distance decay function. For RGSs with multiple entrances, the evacuation distance from the population demand point to the nearest entrance was set as *d*_*kj*_. The distance decay effect assumed that the RGSs near the population centers attract more refugees than far centers within the catchment area. In this study, a Gaussian function was applied to calculate the weight of the population at location *k*:

$$g(d_{kj},d_{o})=\left\{ \begin{array}{c} \frac{e^{-\frac{1}{2}*{\left(\frac{d_{kj}}{d_{o}}\right)}^{2}}-e^{-\frac{1}{2}}}{1-e^{-\frac{1}{2}}},\;d_{kj}\leq d_{o}\\ 0,\;d_{kj}>d_{o} \end{array}\right..$$ (2)

The walking accessibility to RGSs was calculated in multiple scenarios as the main transportation mode after a large earthquake because other modes of transportation may be blocked or destroyed. Based on an investigation of 311 earthquakes in eastern Japan, 44 m/min was selected as the walking evacuation rate [32]. The threshold (d_o_) is a critical factor in the spatial accessibility measurement. After a strong earthquake, the evacuation time can be divided into three response stages: (1) evacuees immediately move from affected areas to on-site shelters within 500 m for protection after an earthquake warning [46]. (2) Secondary damage is easily triggered within 20–30 min after a large earthquake. Evacuees move from on-site shelters to safer shelters, such as short- and medium-term shelters. (3) After termination of the earthquake warning, homeless evacuees move to long-term shelters, where long distances characterize the main evacuation patterns. According to requirements established by the Chinese government, the maximum walking time for an adult evacuee is 1 h. Thus, 10, 20, 30, and 60 min evacuation scenarios were selected to analyze RGS accessibility in Nanjing.

Second, for each demand location (*i*), i.e., all RGS locations (*j*) within the evacuation threshold (*d*_*o*_) from *i* (catchment area *i*), we calculated the sum of the supply-to-demand ratio, *Rj*, within catchment area *i* after using the same distance discount function (*R*_*j*_ was also weighted using a Gaussian equation):

$$A_{i}=\underset{j\in \{d_{ij}\leq d_{o}\}}{\sum }[g(d_{kj},d_{o})R_{j}],$$ (3)

where *A*_*i*_ is the accessibility of the RGS (larger A_i_ values indicate better RGS accessibility). The advantage of Ga2SFCA is that the resulting accessibility value is not an index. Rather, it is a unit that can be considered the per capita occupancy of an GRS at a certain demand point. The unit can be easily interpreted and compared with the minimum required shelter area per capita.

### Gini coefficient and Lorenz curve

The Gini coefficient (GC) and Lorenz curve are widely used in studies on the social equity of public service facilities [47]. Recently, they have been applied to the studies on accessibility [48]. This study used the Lorenz curve and GC to establish possible relationships between the RGS accessibility and population density, which could aid in understanding the distribution of the overall accessibility and demand in the four evacuation time scenarios. The GC was calculated as follows:

$$GC=1-\frac{1}{n}[2\sum_{i=1}^{n-1}a_{i}+1],$$ (4)

where *GC* is the Gini coefficient of the RGS accessibility, *n* is the number of population demand points, *a*_*i*_ is the proportion of the cumulative population demand points with respect to RGS accessibility. The GC ranges from 0 to 1, where 0 represents a perfect match between the RGS accessibility and population demand while, 1 represents a perfect mismatch. Generally, a relatively GC is between 0.3 and 0.4, whereas a GC greater than 0.5 indicates a serious mismatch.

The Lorenz curve is a graphical representation of the GC. In this study, the Lorenz curve was defined as a function between the cumulative percentage of the population and the cumulative percentage of RGS accessibility.

### Bivariate local *Moran’s* I

The bivariate local *Moran’s I* was used to evaluate geographical clustering in the association between the RGS accessibility and population density using the GeoDa software as follows:

$$I_{xy}^{i}=z_{x}^{i}\sum_{j=1}^{n}w_{ij}z_{y}^{j},$$ (5)

where $z_{x}^{i}$ is the normalized value of the RGS accessibility (*x*) of cell *i*, $z_{y}^{j}$ is the normalized value of the population density (*y*) of cell *j*, and *w*_*ij*_ is the spatial weight matrix between cells *i* and j. The spatial relationship between *i* and *j* was divided into five types: high-high cluster (HH), low-low cluster (LL), high-low cluster (HL), low-high cluster (LH), and non-significant. An HH cluster indicates that the cell had a high RGS accessibility and high population density. An LL cluster indicates that the cell had a low RGS accessibility and low population density. The HL cluster indicates that the cell had a high accessibility, but low population density. The LH cluster indicates that the cell had a low accessibility and high population density. The non-significant type indicates that the spatial relationships between the RGS accessibility and population density were not significant.

## Results

### RGS distribution in Nanjing

**Fig 4:**
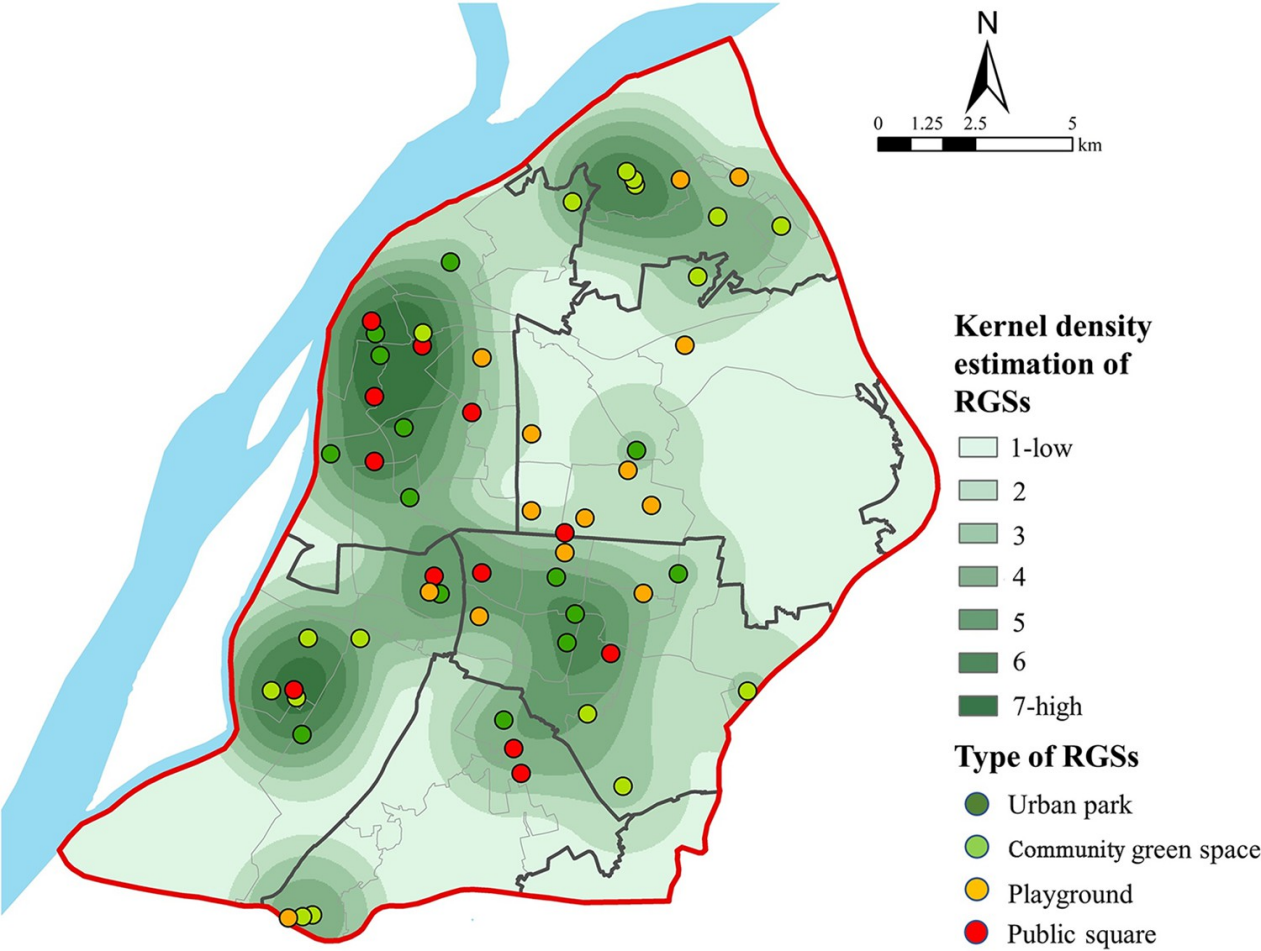
Kernel density estimation of the RGSs.

The total RGS regions of Nanjing were divided into seven levels using the KDE method, with a higher level indicating a higher RGS density (Fig 4). There were significant spatial disparities in the RGS distributions in Nanjing: more RGSs were located in the western regions of the city, such as the Gulou, Jianye, and Qixia districts, which are along the Yangtze River, whereas less were in the Yuhuatai and Xuanwu districts. Approximately 30% of the RGSs were urban parks because the Nanjing government has focused more on urban park development, i.e., the per capita park area is > 15.5 m^2^ within the built-up area. This provides a convenient and facile route for the upgrades and transformation of urban parks to RGSs. The effective refuge areas of RGSs ranged from 1,000 m^2^ to 150 ha, with a mean area of 57,728 m^2^. Only the Yuhuatai district had an RGS of 10.059 m^2^per capita, which already exceeds the essential criterion (1m^2^/person) for emergency shelters in China. However, other districts did not satisfy this criterion (Table 1).

**Table 1:** Comparison of the RGS effective refuge areas in all districts.

District	Population	Effective refuge area of RGS (m^2^)	Per capita effective refuge area of RGS (m^2^/person)
**Xuanwu**	572,572	467,500	0.816
**Gulou**	1,066,400	459,755	0.431
**Qinhuai**	611,057	255,294	0.418
**Jianye**	354,334	197,500	0.557
**Yuhuaitai**	174,200	1,752,160	10.059
**Qixia**	220,273	158,300	0.719

### Spatial distribution of RGS accessibility in four scenarios

**Fig 5:**
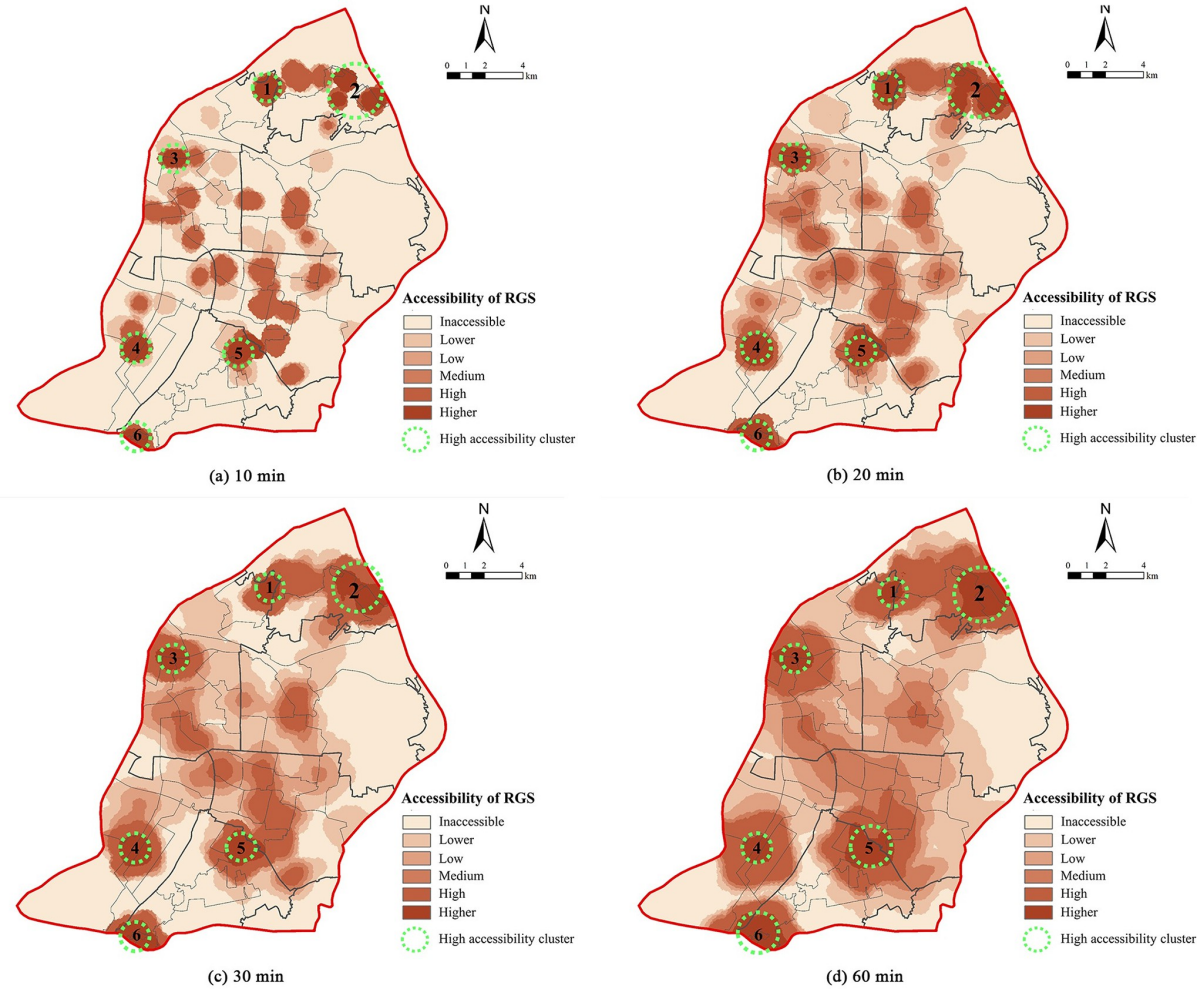
Maps of the RGS accessibility in the four scenarios. The number represents the high accessibility cluster: 1 is Mufu Town, 2 is Dingjia Village, 3 is Xiuqiu Park, 4 is Hexi Central Park, 5 is the Yuhuatai Scenic Spot, and 6 is the Meiling Community.

As the RGSs were not uniformly distributed in Nanjing, an assessment of the accessibility is essential to ensure that citizens from the entire urban area have ample opportunity for RGS access. The RGS accessibility under the four evacuation time scenarios (10, 20, 30, and 60 min) was calculated using the Ga2SFCA method in the ArcGIS 10.6 system. To facilitate a comparison among multiple scenarios, the accessibility results were compared with the minimum per capita RGS (1m^2^/person) [18], and were classified into six levels: inaccessible (< 0.0001), lower accessibility (0.0001–0.1), low accessibility (0.1–0.3), medium accessibility (0.3–0.6), high accessibility (0.6–1.0), and higher accessibility (>1.0).(Fig 5).

In the 10 min scenario, the accessibility distribution was extremely unequal: the average RGS accessibility was 2.567, with a standard deviation (SD) as high as 22.912 (Table 2). Only 6.708% of the cells belonged to the higher or high type, whereas most of the cells (91.82%) belonged to the inaccessible level. In the 20 min scenario, the mean accessibility score and SD decreased to 1.234 and 7.073, respectively. The frequency cells of the inaccessible type decreased by 15.502%, with a corresponding increase in the other types. In the 30 min scenario, with the increase in the threshold, the average accessibility score and SD further decreased to 1.119 and 4.335, respectively. The number of cells with inaccessibility gradually decreased to 2,777. In contrast, the number of medium- and high-accessibility cells increased dramatically to 946. In the 60 min scenario, the mean accessibility score decreased to 0.955, as well as the SD, implying an enhanced spatial agglomeration trend. From the 10 min to 60 min scenario, the inaccessible areas gradually contracted, and the percentage of medium- and high-accessibility cells increased from 7.372 to 32.43%.

**Table 2:** Descriptive statistics of the RGS accessibility under four scenarios.

Scenarios	Accessibility cell frequency						Accessibility score			
	Inaccessible	Lower	Low	Medium	High	Higher	Maximum	Minimum	Mean	SD
**10 min**	4284	33	5	31	87	226	387.027	0.000	2.567	22.912
**20 min**	3561	305	197	141	100	362	131.269	0.000	1.234	7.073
**30 min**	2777	611	332	269	167	510	63.022	0.000	1.119	4.335
**60 min**	1694	864	595	479	195	839	20.446	0.000	0.955	2.224

Although various degrees of spatial disparity in the RGS accessibility always existed in the different evacuation times scenarios, they showed similar spatial patterns (Fig 5). Areas with high or higher accessibility were distributed in the northern and southern regions while most of the urban center was less than medium accessibility. The distribution of cells with higher accessibility was centralized in Mufu Town, Dingjia Village, Xiuqiu Park, Hexi Central Park, and the Yuhuatai scenic spot. These areas were characterized by a high RGS density or large RGS and smaller population areas (Fig 2). Dingjia Village, Meiling Community and Mufu Town were surrounded by several residential green spaces with evacuation functions. Their populations were relatively small, such that the RGSs basically met the demand of the surrounding areas. The Yuhuatai Scenic Spot, Xiuqiu park, and Hexi Central Park had a large shelter space with a strong evacuation service capacity.

The results showed that the RGS accessibility gradually improved with the increase in evacuation time. However, most cells were located at the inaccessibility or low accessibility level in each scenario, indicating that people living in most cells could not access an RGS within 60 min of the evacuation time. Only residents at a distance of a few cells could easily reach their respective RGSs. The per capita RGS of the Yuhuatai district conformed to the essential criteria for emergency shelters in China. Some parts of the Yuhuatai district also characterized by to low or lower accessibility areas (Fig 5 and Table 2). Therefore, it seems that the per capita indicator was not well suitable for measuring the refuge capacity of the RGSs.

### Spatial mismatch between RGS accessibility and population density

**Fig 6:**
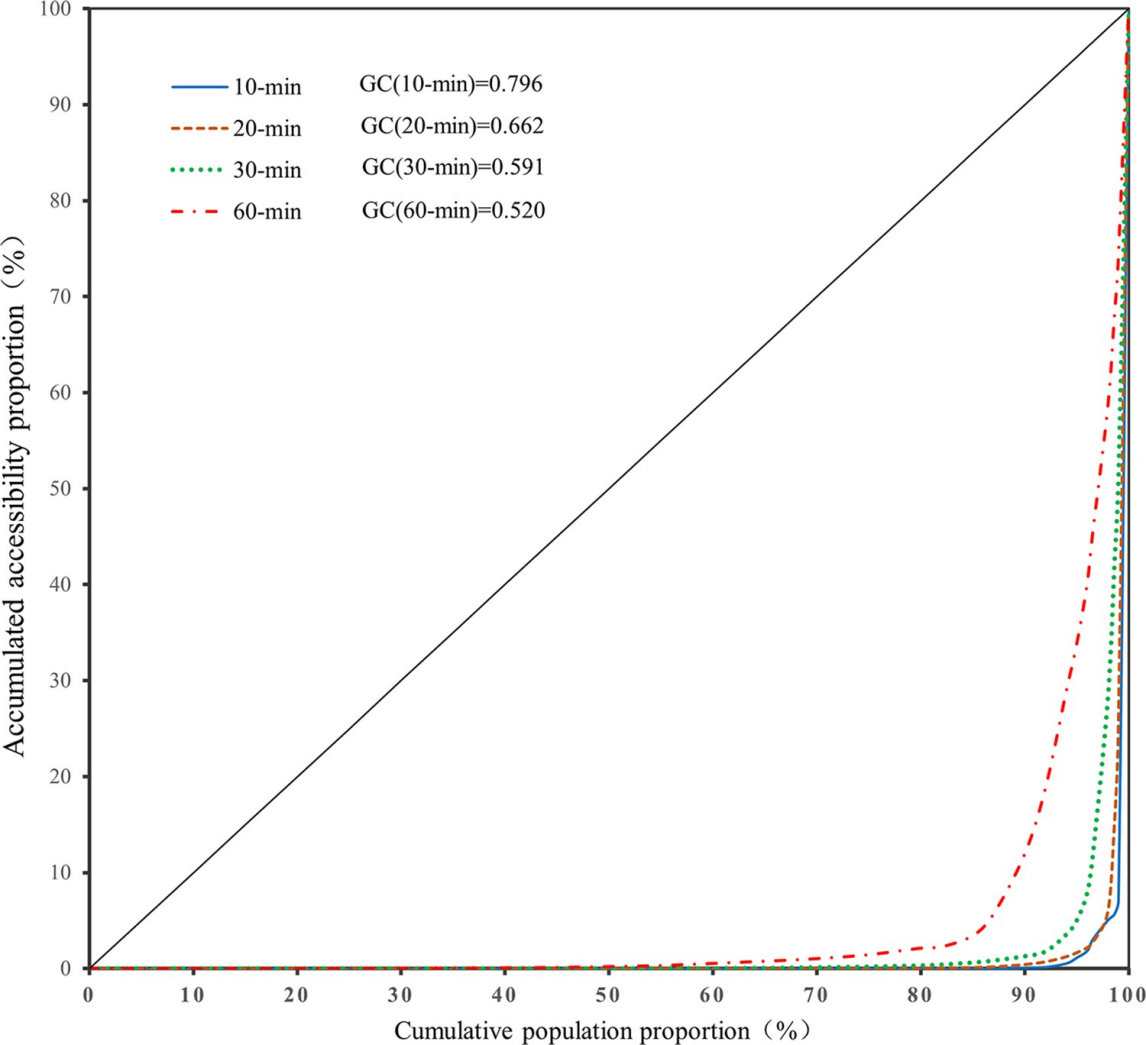
Lorenz curves and GCs of the RGS accessibility.

To identify the spatial disparities between the accessibility and population density, the Lorenz curve and GC were applied to all 4,666 cells in Nanjing. The Lorenz curve and GC provided a satisfactory visual representation of the spatial inequality in term of accessibility [48, 49]. The GC ranged between 0 and 1, where 0 represents perfect equality and 1 represents perfect inequality. As shown in Fig 6, 90% of the population had an RGS accessibility of only 12% in the 60 min scenario, whereas 90% of the population had less than 5% accessibility in the three other scenarios. This analysis showed that most citizens lived in low-accessibility areas. Approximately 90% of the population had relatively few opportunities to access RGS services. The GCs under the four scenarios were 0.796, 0.662, 0.591, and 0.520, respectively, all greater than 0.5, indicating a significant spatial inequality to RGS access among the service population in Nanjing.

**Fig 7:**
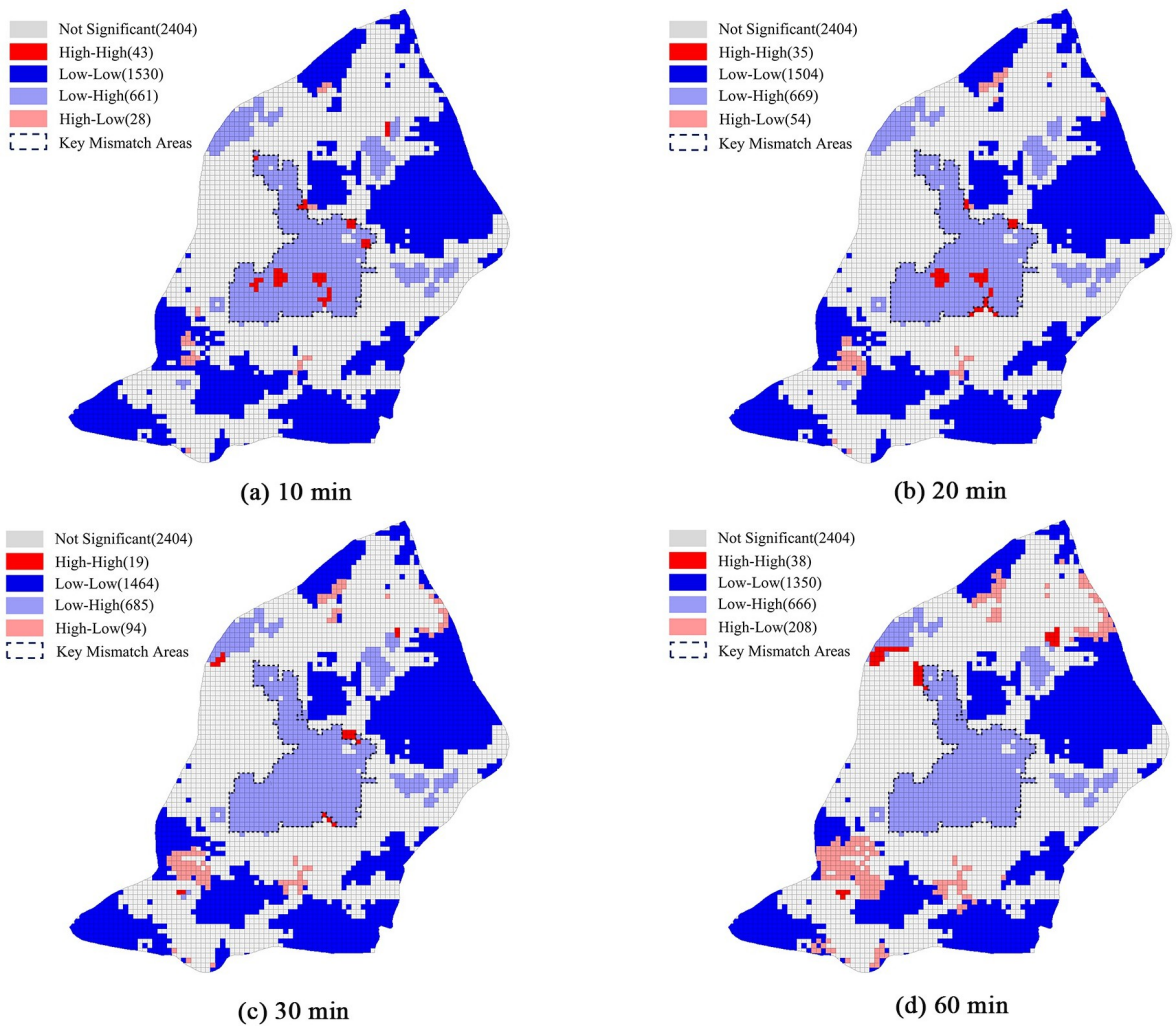
Spatial mismatch maps between the RGS accessibility and population density in the four scenarios.

The bivariate local *Moran’s I* was calculated to further explore the spatial mismatch between RGS accessibility and population density. The spatial relationships were divided into five types (Fig 7): high-high (HH), low-low (LL), low-high (LH), high-low (HL), and non-significant. Both the HH and LL clusters indicated a spatial match between the RGS accessibility and population density, but the LH and HL clusters indicated a spatial mismatch. The LH cluster indicated that the cells with an RGS demand outweighed the supply, whereas the HL cluster indicated that the RGS supply outweighed the demand. The focus of this study was the LH and HL clusters.

In the 10 min scenario, 661 cells belonged to the LH cluster, whereas only 28 cells belonged to the HL cluster. In the 20 and 30 min scenarios, the number of cells belonging to the LH cluster increased to 669 and 685, respectively, while the number of cells belonging to the HL cluster increased to 54 and 94, respectively. In the 60 min scenario, the number of spatial mismatch cells increased significantly. However, the number of cells in the LH cluster decreased to 666, whereas the number of the cells in the HL cluster increased to 208. Consequently, a significant spatial mismatch existed between the RGS accessibility and population density in Nanjing. A more severe spatial mismatch emerged with the increase in evacuation time.

High-High indicated the cell had a high RGS accessibility and high population density, Low-Low indicated a low RGS accessibility and low population density, Low-High indicated a low RGS accessibility and high population density, High-Low indicated a high RGS accessibility and low population density, and Not Significant indicated that the spatial relationships between the RGS accessibility and population density were not significant.

The spatial mismatch patterns between the RGS accessibility and population density in the four scenarios are similar: the underserved regions (LH cluster) were mainly distributed in the center of the urban area while the overserved regions (HL cluster) were dispersed in the northern and southern regions of the urban area. As the population density was relatively high at the center of the urban area, even some subdistricts reached 47,000 person/km^2^, which has led to a substantial number of people sharing limited RGS services (Fig 2). Furthermore, most of the urban center is located in the old town of Nanjing, where a large RGS with high-intensity land development and limited land supply was constructed, resulting in low RGS access.

## Discussion

This study showed that the RGS accessibility distribution varied significantly under the four evacuation time scenarios in Nanjing. However, it exhibited clustering characteristics. High-accessibility spaces were distributed in the northern and southern regions of the city in each scenario. Although the RGS accessibility generally presented an upward trend following the increase in the evacuation time, few regions had high accessibility in the four scenarios, and the current RGSs were only accessible in the 60 min scenario for 10% of the population. This indicated that deficient RGSs and high population density exacerbated the poor accessibility to RGSs in Nanjing. To ensure the safety and efficiency of an evacuation, future urban construction plans must include adequately designed RGSs, especially near densely inhabited districts.

### RGS construction of urbanizing historic city

This study found a serious spatial mismatch between the RGS accessibility and population density in the urban center, where residents cannot easily access RGS services easily. Nanjing has a continuous urban construction history spanning thousands of years, which has therefore formed compact layouts and intensive construction in the urban center, with a consequent reduction in the refuge space. In contrast, most public buildings, retail activities, as well as historic sites and sightseeing attractions are located in the urban center, which has become the most dynamically developing area during the rapid urbanization process, resulting in intense construction and a dense population. The compact urban fabric, rich cultural heritage, and scarcity of land limit the RGS construction, while the high population density requires a large supply of sheltered spaces. This serious discrepancy between the RGS supply and demand, not only exists in Nanjing, but also in many other rapidly developing historic city centers. A similar phenomenon was found in Italy, where the main refuge facilities are commonly located outside of historic city centers [4]. Damage due to building collapse or fire can be more serious in historic city centers, which have a high population density, crowded streets, and more old buildings with no safety designs. Therefore, increasing the RGS provisions in urban centers is a substantial challenge for rapidly urbanizing historic cities.

Achieving the increased utilization of undeveloped open space to build new RGS, like that in new development cities is more complex in historic cities. Urban green spaces, including urban parks, green buffer zones, squares, attached green spaces, and suburban parks spaces, can easily transform into various RGSs after an earthquake occurs owing to their spatial layout, planar form, and infrastructure. For instance, urban parks with areas of 1,000 m^2^ to over 3 ha are optimally suited as temporary RGSs by adapting emergency facilities. Large-scale urban and suburban parks are suitable for use as long-term RGSs [10]. The construction of RGSs based on existing urban green spaces can support post-earthquake evacuations on limited urban land, thus allowing for the preservation of the existing urban form and neighborhood characteristics [50]. Nanjing was one of the earliest cities to establish a complete urban green space system in China. Planners and policymakers can increase the supply and enlarge the catchment areas of RGSs based on the urban green space, availability which can improve the spatial accessibility to the RGSs in Nanjing.

### Community green space potential for in-place shelters

The results from this study also showed that RGS accessibility in the 10 min scenario was at the lowest level. More than 90% of the population could not access an RGS. Since the construction of an RGS is a government achievement, most governments prefer to establish high-level or long-term emergency shelters, which results in a limited supply of in-place RGSs. After an earthquake, almost all of the refugees chose in-place shelters within a 10 min walking distance [7]. Historic cities, especially in their central areas, have limited green spaces for conversion to large refuge spaces. Masuda [10] notes that community open spaces (e.g., community parks, residential green spaces, and neighborhood green spaces) with areas of 500–2,000 m^2^ are immediately available as in-place shelters in Japan. In this study, there was no large RGS around the Meiling Community, Mufu Town, or Dingjia Village. However, the accessibility in the 10 min evacuation scenario was high because there were several community-based RGSs appropriate for the target population. RGSs consist mainly of urban parks in Nanjing, which ignores the emergency evacuation potential of community green spaces.

**Fig 8:**
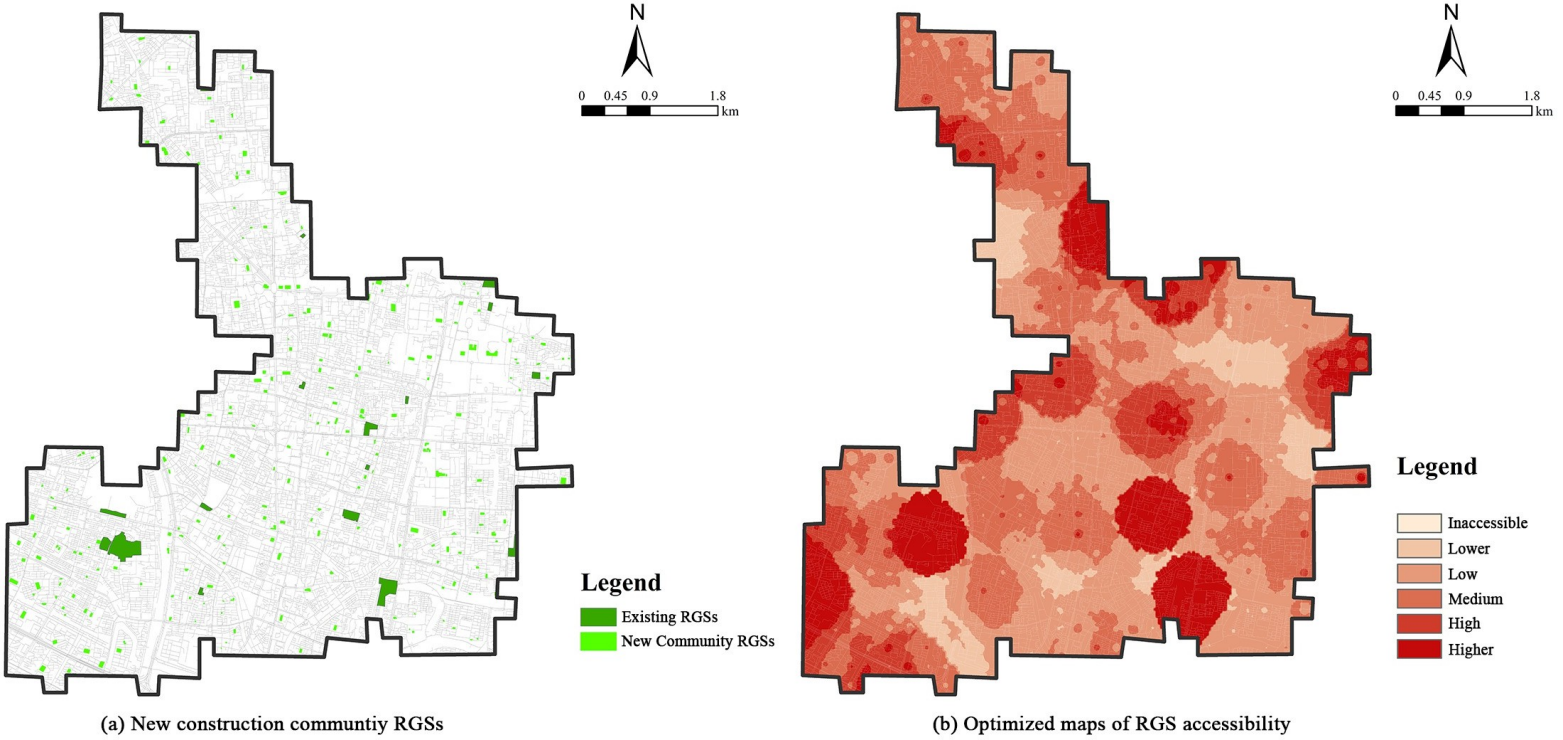
Optimized maps of RGS accessibility in 10 min scenarios.

Using the key mismatch areas identified in this study as examples, we selected community green spaces with evacuation potential and calculated the RGS accessibility under the 10 min scenario. A total of 234 community green spaces were selected, which were located within and around residential areas, away from geological hazard and disaster potential areas, with relatively flat and open terrain. Most of these community green spaces were less than 2,000 m^2^ (Fig 8A). The optimized refuge area was approximately twice as large as current RGSs (Table 3). The 10 min RGS accessibility significantly improved after optimization (Fig 8B). In terms of the overall accessibility, the average accessibility after optimization significantly improved from 0.19 to 0.60 and was more evenly distributed. In terms of the accessibility range, unreachable cells decreased from 525 to 192, i.e., the inaccessible area reduced from 97.77 to 35.75%. The above-medium accessible area increased from 1.86 to 35.01%. In terms of the accessible population, only 30% of the population had access to exiting RGSs, which increased to 69.82% after optimization (Table 3, Fig 8B). The conversion of community green spaces to shelters eliminated blind spots during emergency evacuation caused by the high population density and insufficient RGSs, thus significantly relieving the pressure associated with in-place evacuations. This also exhibited the in-place shelter potential of community green spaces. Therefore, integrating community green space, such as community parks, community playgrounds, and mini green spaces, into evacuation and shelter planning should be encouraged in historic urban areas. In addition, the proposed community RGSs could provide a direct reference for the next step of improving the refuge capacity of Nanjing.

**Table 3:** Descriptive statistics of RGS accessibility in 10 min scenario.

	Total area of GRS (m^2^)	Accessible population percentage (%)	Accessibility score		Accessibility cell frequency					
			Mean	SD	Inaccessible	Lower	Low	Medium	High	Higher
**Existing RGSs**	320,911.34	30.00	0.19	2.71	525	1	1	5	1	4
**Optimized RGSs**	649,053.89	69.82	0.60	2.58	192	77	80	98	36	54

### Study limitations

This study had several limitations. First, we assumed that the road network was not destroyed after a hypothetical earthquake, and walking was the only transportation mode. However, a Japanese study showed that approximately half of the cars selected long-distance evacuation while approximately one-third were in a traffic jam after an earthquake [32]. Therefore, the RGS accessibility based on the Ga2SFCA method should be explored by considering road closures with varying transportation modes in future studies. Second, although we measured the RGS accessibility at multi-spatial scales, there were overlap in RGS usage, which is the optimal scale to provide a better guidance for the practice deserves further study. Third, the RGS accessibility was closely related to the number and location of entrances. For RGSs with multiple entrances, this study selected the nearest entrance to each population demand point to calculate the catchment area, which improved the calculation accuracy. In fact, the influence of entrances on accessibility was complex. This present study did not analyze this influence, which is an important topic for future research.

## Conclusion

Historic cities have formed a dense and organic urban structure after centuries-long development. Intense urbanization processes have led to an increase in the population density and demand for evacuation strategies. Evaluations of whether the spatial distribution of RGSs is reasonable and whether the RGS evacuation capacity meets demand are key to building urban safety nets in rapidly urbanizing historic cities. In this study, the Ga2SFCA and bivariate local *Moran’s I* methods were used to measure the spatial distribution of RGS accessibility and identify the spatial mismatch between the RGS accessibility and population density in Nanjing. We showed that high-accessibility spaces were mainly distributed in the northern and southern regions of the city. Underserved areas of RGSs were predominantly located in urban centers. We also showed that with the increase in the evacuation time, although the accessibility indicators for the RGSs improved under the four scenarios, the RGSs could not sufficiently satisfy the emergency shelter demands of Nanjing citizens. The RGS accessibility in the 10 min scenario was at the lowest level. Most of the population could not access an RGS. Given the specificity in the fabric of historic cities, this study suggests that the number of RGSs in underserved areas should be appropriately increased based on the availability and accessibility of exiting green spaces, especially community RGSs, to improve the safety net of Nanjing residents. Compared with other studies that specifically focus on RGS accessibility, this paper focused on RGS construction in historic cities characterized by rapid urbanization. The results of this study not only provide important guideline for the optimization of the regional layout of RGSs in Nanjing, but can also provide a strong reference for the construction of safe environment in other rapidly developing historic cities around the world.

## Supporting information

S1 Table Data sources. (DOCX)

S2 Table Results of RGS accessibility in four scenarios. (XLSX)

S3 Table Results of optimized RGS accessibility in the key mismatch areas under 10 min scenario. (XLSX)

S1 Fig Maps of existing RGS accessibility in the key mismatch areas under 10 min scenario. (TIF)
