# Supplementary material for: Measuring spatial accessibility to refuge green space after earthquakes: A case study of Nanjing, China
Source: PLoS One. 2022 Jun 28;17(6):e0270035. doi: 10.1371/journal.pone.0270035 (PMC9239463; doi:10.1371/journal.pone.0270035)
Supplement: S1 Table — (DOCX) [file pone.0270035.s001.docx]

# S1 Appendix. Data sources

| **Data** | **Data source** |
| --- | --- |
| Refuge green space data | <http://yjzh.njyjgl.cn:8000/#/> |
| Road network | https://www.openstreetmap.org |
| District and sub-district data | https://www.resdc.cn/Default.aspx |
| Population data | <http://tjj.nanjing.gov.cn/material/njnj_2020/renkou/index.htm>, Xuanwu Statistical Yearbook 2020, Qinhuai Statistical Yearbook 2020, Yuhua Statistical Yearbook 2020, Gulou Statistical Yearbook 2020, Qixia Statistical Yearbook2020, Jianye Statistical Yearbook 2020 |
| Building density | http://www.locaspace.cn/ |
