# Supplementary figures and images for: Measuring spatial accessibility to refuge green space after earthquakes: A case study of Nanjing, China
Source: PLoS One. 2022 Jun 28;17(6):e0270035. doi: 10.1371/journal.pone.0270035 (PMC9239463; doi:10.1371/journal.pone.0270035)

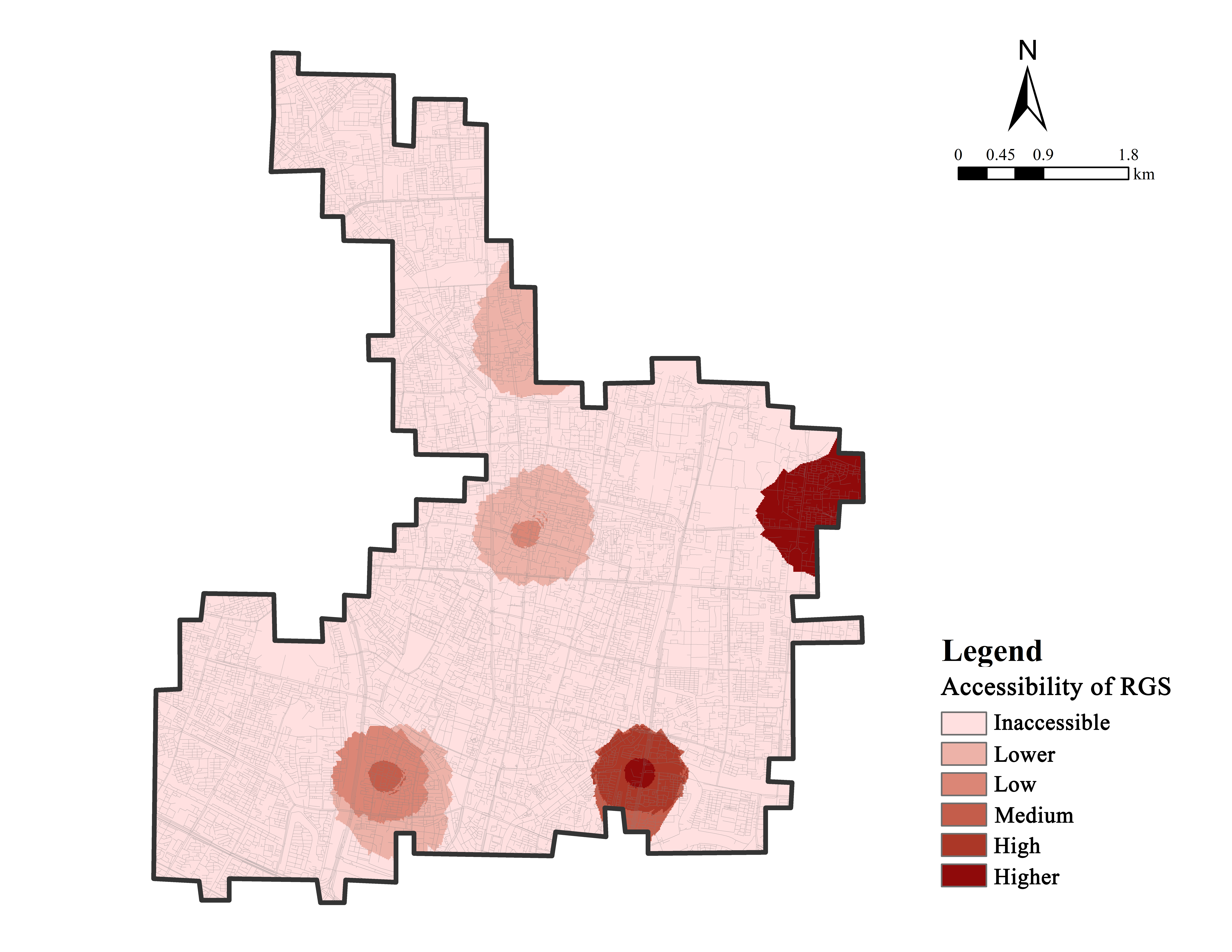

Supplement: S1 Fig — (TIF) [file pone.0270035.s004.tif]
